# Near-infrared hyperspectral imaging to map collagen content in prehistoric bones for radiocarbon dating

**DOI:** 10.1038/s42004-023-00848-y

**Published:** 2023-04-11

**Authors:** Cristina Malegori, Giorgia Sciutto, Paolo Oliveri, Silvia Prati, Lucrezia Gatti, Emilio Catelli, Stefano Benazzi, Silvia Cercatillo, Dragana Paleček, Rocco Mazzeo, Sahra Talamo

**Affiliations:** 1 Department of Pharmacy, University of Genova, Viale Cembrano 4, I-16148 Genova, Italy; 2 University of Bologna, Department of Chemistry “G. Ciamician”, Ravenna Campus, Via Guaccimanni, 42, 48121 Ravenna, Italy; 3 Department of Cultural Heritage, University of Bologna, Via degli Ariani 1, 48121 Ravenna, Italy; 4 Department of Chemistry G. Ciamician, Alma Mater Studiorum, University of Bologna, Via Selmi 2, 40126 Bologna, Italy

**Keywords:** Imaging studies, Infrared spectroscopy, Mass spectrometry

## Abstract

Many of the rarest prehistoric bones found by archaeologists are enormously precious and are considered to be part of our cultural and historical patrimony. Radiocarbon dating is a well-established technique that estimates the ages of bones by analysing the collagen still present. However, this method is destructive, and its use must be limited. In this study, we used imaging technology to quantify the presence of collagen in bone samples in a non-destructive way to select the most suitable samples (or sample regions) to be submitted to radiocarbon dating analysis. Near-infrared spectroscopy (NIR) that was connected to a camera with hyperspectral imaging (HSI) was used along with a chemometric model to create chemical images of the distribution of collagen in ancient bones. This model quantifies the collagen at every pixel and thus provides a chemical mapping of collagen content. Our results will offer significant advances for the study of human evolution as we will be able to minimise the destruction of valuable bone material, which is under the protection and enhancement of European cultural heritage and thus allow us to contextualise the valuable object by providing an accurate calendar age.

## Abstract (web summary)

The collagen present in rare prehistoric bones allows for their age to be estimated by radiocarbon dating, but this method is destructive towards these precious archaeological remains. Here, the authors report a non-destructive method based on near-infrared hyperspectral imaging to precisely localize the collagen preserved in parts of ancient specimens suitable for radiocarbon dating.

## Introduction

Radiocarbon (^14^C) dating of bones has contributed significantly to understanding human prehistory. In fact, understanding the spread of *Homo sapiens* in waves across much of the world, from Africa to Europe, its encounter with other human lineages (Neanderthals and Denisovans) and its ultimate success in populating the entire planet, is one of the main goals of Palaeolithic archaeology. Moreover, investigations over the past decade have revealed complex scenarios of hominin migrations, interbreeding and extinctions^1–4^.

One factor that characterises the complexity of these studies is that archaeological bones are a sort of living memory. Bones can provide a great deal of information about ancient populations’ lives: what they ate, their reproductive habits, their diseases and the migrations they undertook. However, bones cannot give us all the information we so covet. Their potential to convey information is limited by how much collagen is preserved in them. Collagen is the protein we must extract to perform a radiocarbon analysis.

The good news, however, is that even sites with poor collagen preservation may have some bones that retain a fair amount of collagen or at least enough to perform a ^14^C analysis. However, it is extremely difficult, costly and time consuming to analyse all the bones present at one archaeological site for collagen preservation, as it would entail high costs for pretreatment in the ^14^C lab, and analysis itself. Most importantly, it would result in the destruction of valuable material. In fact, sampling for ^14^C dating is a destructive process, and human fossils and/or bone artefacts are increasingly rarer and more precious over time. Because of the diagenetic alteration of collagen over time, large starting weights of Palaeolithic bones (≥500 mg bone material) are necessary to extract sufficient collagen for accelerator mass spectrometry (AMS) ^14^C dating (minimum 1% yield)^5^. Moreover, many of the most precious archaeological bones are too small (<200 mg of bone material) and/or too beautiful for sampling. Therefore, obtaining preliminary, non-destructive information about the distribution of collagen on a bone sample is crucial.

It is in this context that the technique described here really shines because it allows us to obtain information both on the location and on the content of the collagen still present in a bone sample. The near-infrared hyperspectral imaging camera (NIR-HSI) used in the present study is a line-scan (push-broom) system that acquires chemical images in which, for every pixel, a full spectrum in the 1000–2500 nm spectral range (near infrared) is recorded. NIR-HSI analysis is completely non-destructive. The time required for the analysis of a single bone sample is of few minutes and, therefore, the system can examine many samples in a single day to find those suitable for analysis, saving time and money and the unnecessary waste of valuable material, greatly reducing time, costs and destruction of valuable samples. Therefore, it is expected that this technique will support the selection of samples to be submitted to radiocarbon analysis at many sites where previous attempts have not been possible because of poor preservation. In the last decades, several applications have been proposed to prescreen archaeological bones to investigate the presence of collagen before attempting costly radiocarbon dating.

Chemical indicators (e.g., the collagen yield, the %N and %C content, the derived C:N ratio and δ^15^N and δ^13^C values) are routinely used to indicate the quality and overall preservation of bone collagen^5–7^. At least a 1% weight of collagen is considered necessary, and samples of lower yield are potentially problematic^5^. The C:N ratio should fall between 2.9 and 3.6, as occurs in modern animals and humans^5,7^. However, the analysis of whole bone percent nitrogen (%N) is the preferred form of bone prescreening in the radiocarbon community^8–10^. This method requires the preparation of only 5 mg of bone material, but it still involves bone destruction, albeit small. The 0.70% N threshold for Palaeolithic samples correctly predicts the state of bone preservation, and >1% collagen by weight^8^ is usually an indication of good collagen preservation, as mentioned above.

A more recent trend, which is not as invasive as the %N determination, is the application of Fourier Transform Infrared (FTIR) Spectroscopy and Raman spectroscopy to characterise collagen in archaeological bones^11–16^. Both techniques provide the analyst with complementary molecular information, related to vibrational modes of chemical bonds. However, methods usually allow to measure in the midIR range (2500 nm–25,000 nm)^17^ with limitations intrinsically connected to this spectral range and Raman spectroscopy uses a laser at a particular exposure to measure wavelength shifts in scattered light^18^. Even if both techniques are potentially non-destructive screening methods and provide a suitable tool for collagen analysis^19,20^, both presents some drawback, if compared with near-infrared spectroscopy. Raman, in particular, uses a laser, which may damage the surface, degrading the materials and hindering the possibility of submitting the investigated area for further analysis. Moreover, fluorescence side effects may affect the quality of spectral information. NIR spectroscopy presents several advantages related to the cost of instrumentation, the time required for the measurement and the possibility of obtaining information beyond the surface thanks to the penetration depth of NIR radiation^21^.

Recently, single-point NIR spectroscopy has been used to prescreen archaeological bones with the support of appropriate multivariate data analysis. In particular, the NIR spectrometer was used to classify bones from the Holocene to the Late Pleistocene age according to their state of preservation and to quantify the percentage of collagen in both bone powder and whole bone^21^. Appropriate multivariate data analysis was performed on the collected NIR signals, applying established approaches to build predictive models based on discriminant classification (PLS-DA) and quantification (PLS regression). All steps of this chemometric strategy were performed to confirm the reliability of the NIR spectrometer to predict the presence of collagen in bone quickly, non-destructively and with high accuracy^21,22^. However, all the previous methods allowed a selection among the samples, but they could not provide specific information on the most suitable sampling point inside a specimen.

Recently, NIR-HSI has been applied to obtain spectral maps that reveal the collagen distribution in large bones^23^. However, this method offers only an estimation of the distribution of collagen. Moreover, the pretreatment used in this study was not the same as the one used in radiocarbon laboratories^5,24^.

**Fig. 1:**
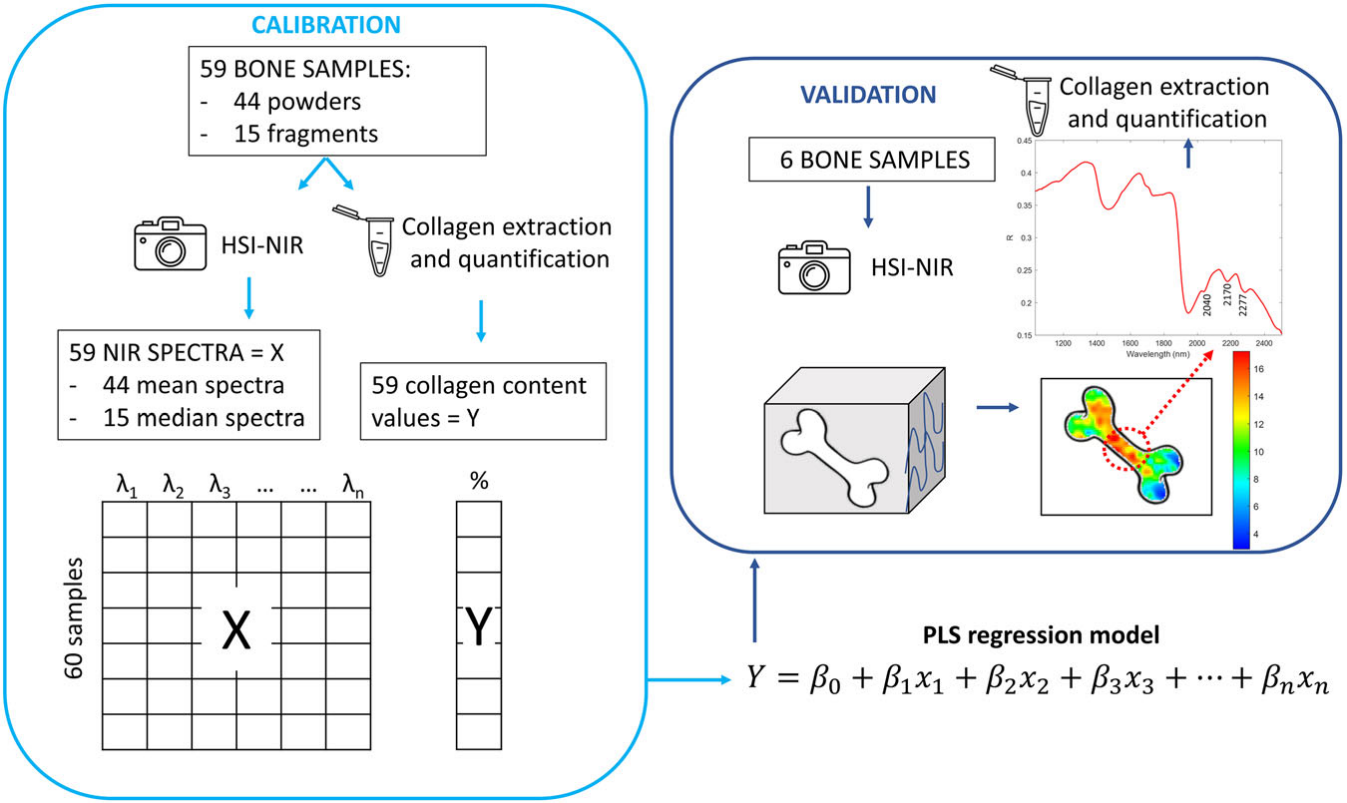
The experimental protocol. The experimental protocol was divided into two parts, the calibration (light blue square) and the validation (dark blue square).

In our study, we built a multivariate regression model using the partial least squares (PLS) method, aiming to map and quantify collagen content in bone samples, thanks to correlations between the relative collagen amount and NIR absorptions, as shown in Fig. 1.

To do this, a regression model was created using the NIR spectra as predictor variables and the collagen content as the response variable. Moreover, we used the updated pretreatment method adopted in Talamo et al.^5^, employing the ultrafilter, to extract collagen for the subsequent ^14^C analysis to verify the predicted collagen content.

The results of our study document how the application of NIR-HSI is a crucial step forward for selecting the sampling point for ^14^C dating, proposing a robust predictive model for obtaining reliable and quantitative information on the distribution of collagen on a single bone specimen. This allows not only selecting the best specimens but also choosing the sampling point in the selected ones based on the amount of collagen predicted. This method helps to drastically reduce the number of samples destroyed for ^14^C analysis, and within the bone, it helps to avoid the selection of areas that may present a quantity of collagen not sufficient for the dating. This increases the preservation of precious archaeological materials.

## Results

In total, 44 archaeological bone samples, including some more than 50,000 years old, were submitted to NIR-HSI analysis and used to obtain quantitative data (i.e., collagen content, determined through collagen extraction, as described in the “Methods” section) in order to build and validate the model (Supplementary Data 1). Those data reflect the real amount of collagen in a bone while considering the up-to-date pretreatment required for radiocarbon dating.

Indeed, NIR-HSI provides the analyst with valuable information to detect collagen, as it is confirmed in the spectrum reported in Fig. 1 and obtained from a bone sample, in which vibration bands related to collagen are clearly identifiable in the spectral region between 2000 and 2300 nm. In more detail, 2040 nm (N-H combination of stretching and deformation mode), 2170 nm (combination band of the first overtone of carbonyl stretching and N-H bending) and 2270 nm (C–H stretching and bending combination band)^21,25^.

A portion of each 44 samples was drilled to obtain powder for subsequent collagen extraction, and of these, only 15 samples were cut and pretreated as fragments. Four of the 44 powder samples were pretreated without the ultrafiltration step (Supplementary Data 1) for a total of 63 analyses.

### Prediction of the model

The results of the regression model presented a root mean square error in cross-validation (RMSECV) of 2.2%. It should be noted that root mean square errors have the statistical meaning of a standard deviation (dispersion parameter), expressed with the same measurement unit and scale as the response variable. Several statistical tests were conducted to evaluate the robustness of the model, such as randomisation and permutation tests, demonstrating the reliability of the collagen concentration estimates obtained using the regression model. Indeed, even by randomising the samples, the error was around 2.2%, while the estimated error by permuting the block of X and Y was significantly higher (about 6%) than the model error. We also report, for each averaged value of the predicted collagen concentration, the standard deviation, which indicates the variability of the collagen distribution in the bone samples. The optimised PLS model was applied to a validation set of six bone samples (described in the following two sections) not used for developing and optimising the model. Three of them (detailed in the “Validation phase with previously extracted collagen” section) were used to recheck the percentage of collagen from bones in which pretreatment for collagen extraction for ^14^C dating had already been performed on powder samples. The other three (detailed in the “Validation phase with subsequently extracted collagen” section) performed pretreatment of the protocol for ^14^C dating, but only after the PLS model predicted precisely where to cut the sample. The prediction was performed at the pixel level, meaning that a collagen value was computed for every image spectrum (point). To visualise the results, false-colour maps were obtained, ranging from blue (minimum collagen content) to red (maximum collagen content).

### Validation phase with previously extracted collagen

**Fig. 2:**
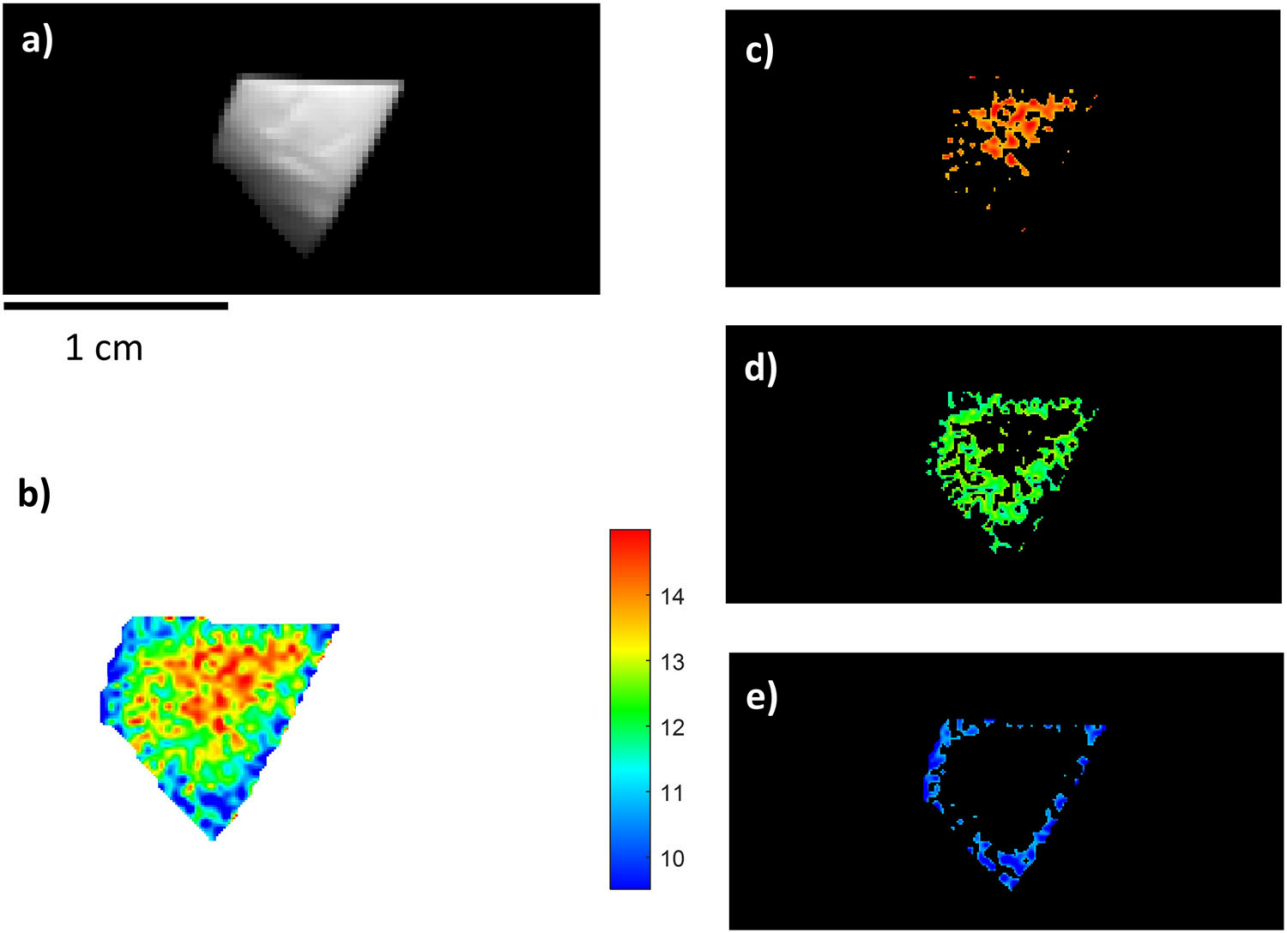
NIR-HSI results for sample BRA-3586. **a** Grey-scale total intensity image; **b** PLS map of the reference samples. The colour bar numbers refer to collagen % predicted by the PLS model; **c**–**e** ROIs referring to areas with different collagen concentrations (blue: low, green: mid-level, red: high) on an individually scaled maps.

The collagen of three animal bone samples had been extracted from powder, using the reported method of Talamo et al.^5^, so the yield (%) of collagen was known. These were subjected to NIR-HIS analysis to assess the prediction ability of the proposed approach. Sample BRA-3586 (Table 1 and Fig. 2) had a good preservation state, based on the amount of collagen predicted, and the PLS model indicated a mean value of 12.37% and a standard deviation of 1.23% in this sample. As previously mentioned, the standard deviation refers to the variability of the collagen distributed in the bone samples. The predicted collagen content fully agreed with the collagen percentages obtained from powder that was previously extracted (12.13% Table 1). The quantitative chemical map of the BRA-3586 sample revealed an inhomogeneous distribution of collagen despite the relatively small sample size. There was a higher concentration in the central part of the sample (with a maximum amount of about 15%), while the surrounding areas had a lower content (with a minimum of about 8%) (Fig. 2).

**Table 1:** Samples used for validation.

BRAVHO lab code	Country-Territory	Sample analysed	Sample taken (mg)	Collagen extracted (mg) >30 kDa	% Collagen >30 kDa	Collagen extracted (mg) <30 KDa	% Collagen <30 KDa	Total collagen % extracted	% Estimation by NIR-HSI fragment
BRA-3586	Central Europe	Powder	479	52.8	11%	5.3	1%	12%	12 ± 2%
BRA-3813	Iberian Peninsula	Powder	799	2.3	0.29%	6.6	0.83%	1%	2 ± 2%
BRA-3607	Iberian Peninsula	Powder	571	78.3	14%	2.5	0%	14%	12 ± 2%
BRA-3367	Central Europe	Piece	496	34.8	7%	10.8	2%	9%	10 ± 2%
BRA-6334	Italian Peninsula	Piece	975	0%	0%	0	0%	0%	2 ± 2%
BRA-6335	Italian Peninsula	Piece	1143	0%	0%	0	0%	0%	2 ± 2%

The six samples used to validate the model with previously extracted collagen in the powder samples and subsequently extracted collagen from the pieces.

**Fig. 3:**
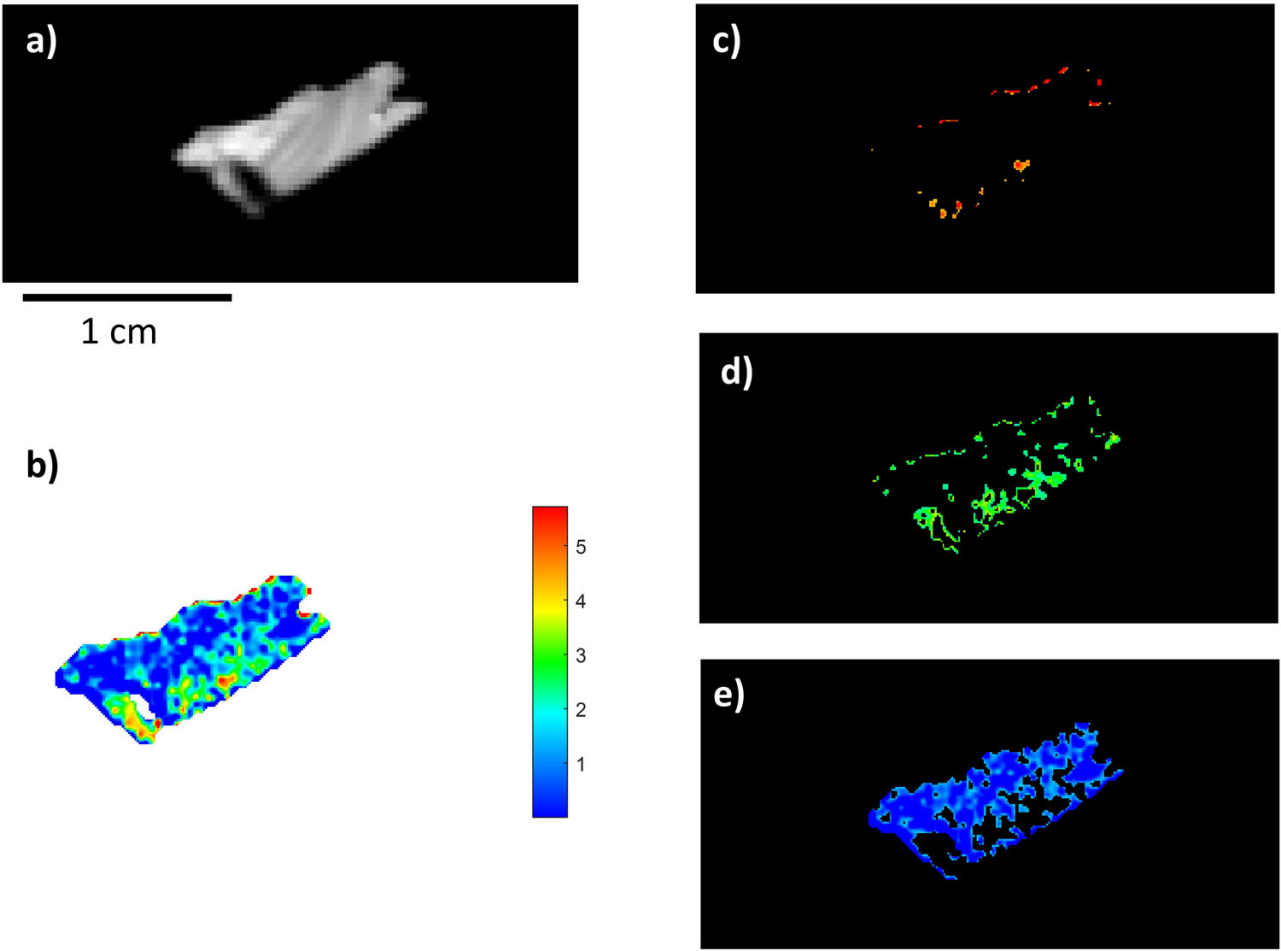
NIR-HSI results for sample BRA-3813. **a** Grey-scale total intensity image; **b** PLS map of the reference samples. The colour bar numbers refer to collagen % predicted by the PLS model; **c**–**e** ROIs referring to areas with different collagen concentrations (blue: low, green: mid-level, red: high) on an individually scaled map.

The second sample (BRA-3813) was known to have reduced collagen content (Table 1), possibly because of an aggressive conservation environment that induced severe diagenetic phenomena, leading to collagen hydrolysis and consequent peptide bond breakage^26,27^. This sample (BRA-3813, Fig. 3) was also used to evaluate the model’s performance. The bone showed an overall low concentration of collagen, and the quantification predicted by the PLS regression model was about 1.61%. This was in accordance with the quantity obtained by extraction from powder (1.12%) using the collagen extraction method in Talamo et al.^5^, and its RMSECV was 2.2%. The sample had a widespread low collagen content (blue and green pixels, ranging between 2 and 0%). A few very limited points had amounts of up to 5%, and their distribution made it difficult to identify an appropriate sampling area.

**Fig. 4:**
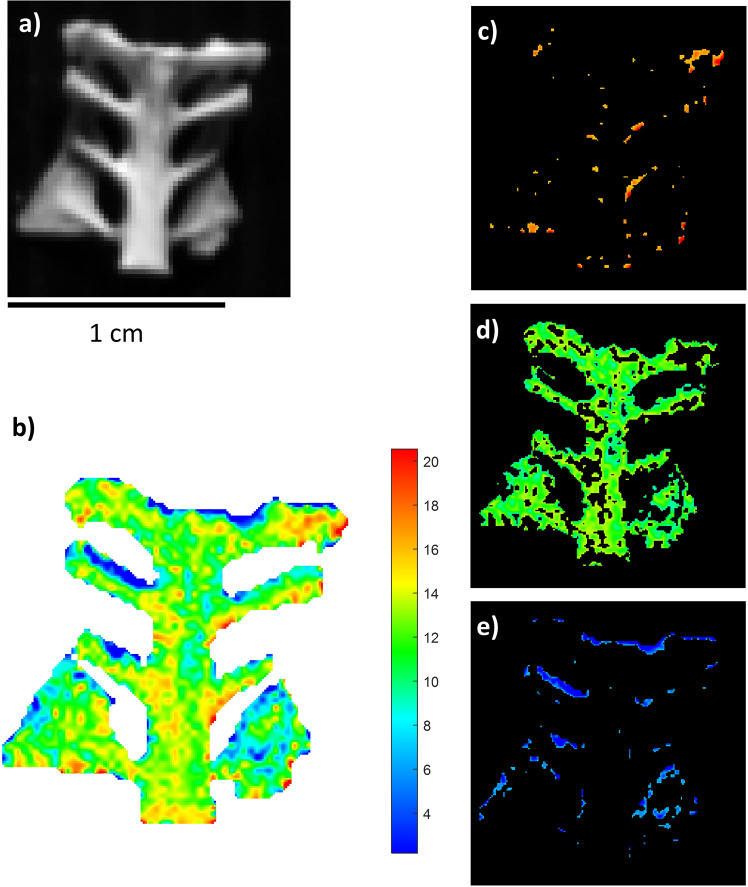
NIR-HSI results for sample BRA-3607. **a** Grey-scale total intensity image; **b** PLS map of the reference samples. The colour bar numbers refer to collagen % predicted by the PLS model; **c**–**e** ROIs referring to areas with different collagen concentrations (blue: low, green: mid-level, red: high) on an individually scaled map.

The third sample (BRA-3607, Fig. 4) had a high collagen content (on powder, it was 14.15%, Table 1), which was expected since it was a relatively modern bird sample. Bands are assigned to a protein compound in all the NIR spectra extracted from the three regions of interest (ROIs). The predicted mean value was about 11.80%, with a standard deviation of 2.75%. The relatively high standard deviation can be justified by a particularly inhomogeneous distribution of collagen, which varied significantly from point to point, as clearly revealed by the quantitative distribution of the PLS map. The highest collagen concentrations were mainly in the conjunctions between the vertebral column and ribs, which were probably more protected from the environment. They had maximum values of around 20%. Despite the relatively recent period of the sample (found on the beach in the Iberian Peninsula), it had a localised decrease of collagen (e.g., in the thinnest and exposed areas), where it was possible to estimate collagen content between 4 and 9%. Overall, the amounts of collagen predicted fell within the range of collagen previously extracted.

### Validation phase with subsequently extracted collagen

To further demonstrate the advantage and the possible real application of this approach to prescreen for selective collagen extraction, three additional samples were selected for the extraction of collagen for radiocarbon dating from the precise area of highest collagen preservation (Table 1).

One of the three samples came from an archaeological site in central Europe, where collagen abundance had been attested by the extraction of different bones at the site but not yet published. Two of the three samples (BRA-6334 and BRA-6335) were from an archaeological site in the Italian peninsula^28^ where the scarce preservation of collagen is well documented.

**Fig. 5:**
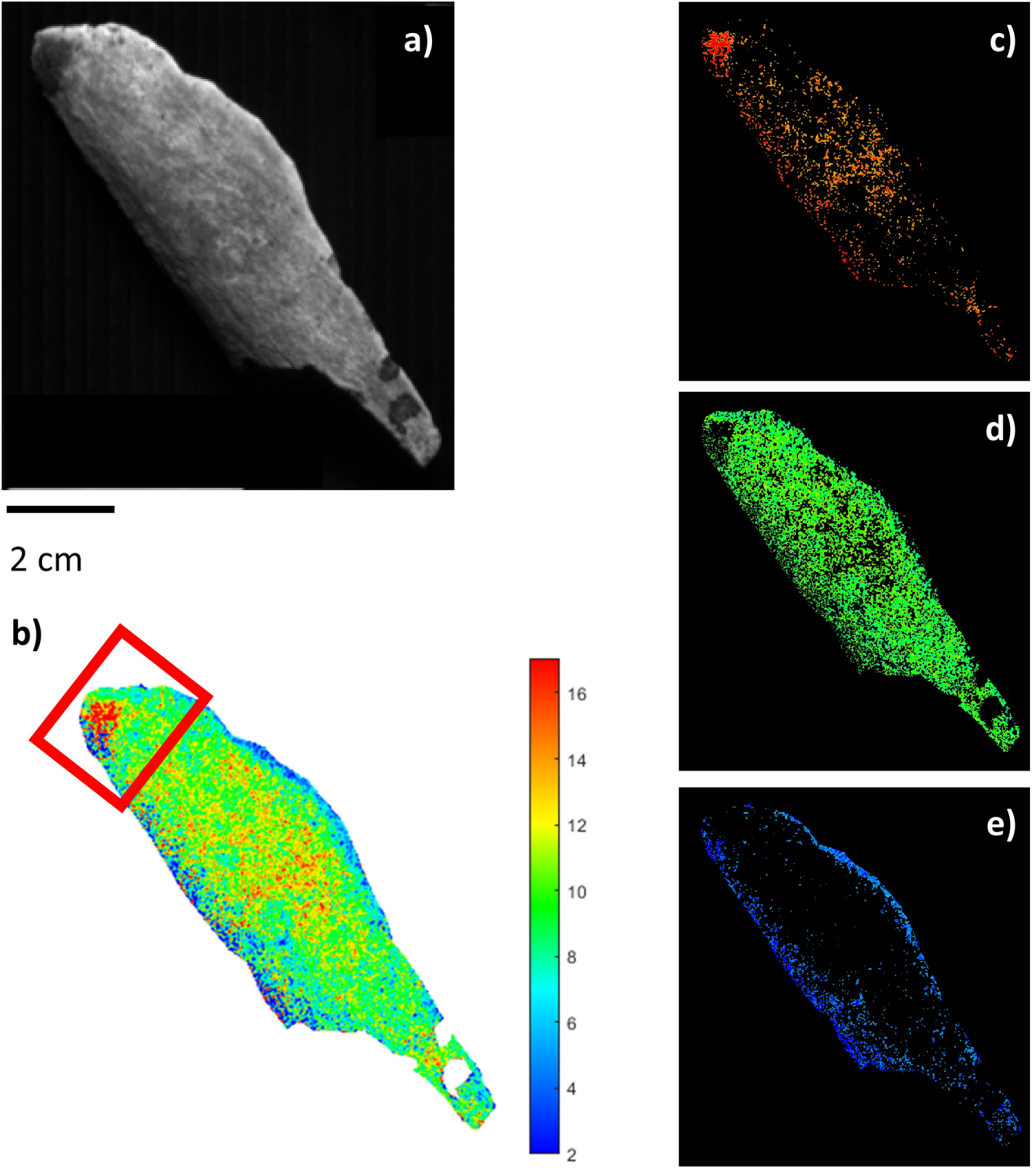
NIR-HSI results for sample BRA-3367. **a** Grey-scale total intensity image; **b** PLS map of the reference samples. The colour bar numbers refer to collagen % predicted by the PLS model. The red square area is the selected sample taken for the extraction of collagen; **c**–**e** ROIs referring to areas with different collagen concentrations (blue: low, green: mid-level, red: high) on an individually scaled map.

The first sample (BRA-3367) showed a quite homogenous distribution and a concentration with a predicted mean value of 9.66% ± 2.65% (Table 1 and Fig. 5).

The fragment in the basal part of the sample (red square in Fig. 5) was cut for the extraction and quantification of collagen. It is worth remembering that predictions were based on the total amount of collagen in a sample. For radiocarbon, we used the ultrafiltration step, which divided the long collagen chain (>30 KDa) from the shorter chain (<30 KDa)^5^. In this case, the collagen was 7% in the long chain and 2.2% in the short one, for a total of 9.2% of collagen extracted for the radiocarbon analysis. This result confirmed the reliability of the prediction.

The other two samples (BRA-6334 and BRA-6335) showed evident concretions on their surfaces coming from the excavated soil and related to this specific site’s environmental and geological conditions. To overcome this drawback, a specific masking strategy was used to isolate the pixels affected by the concretions, submitting only the clean areas for the PLS regression.

**Fig. 6:**
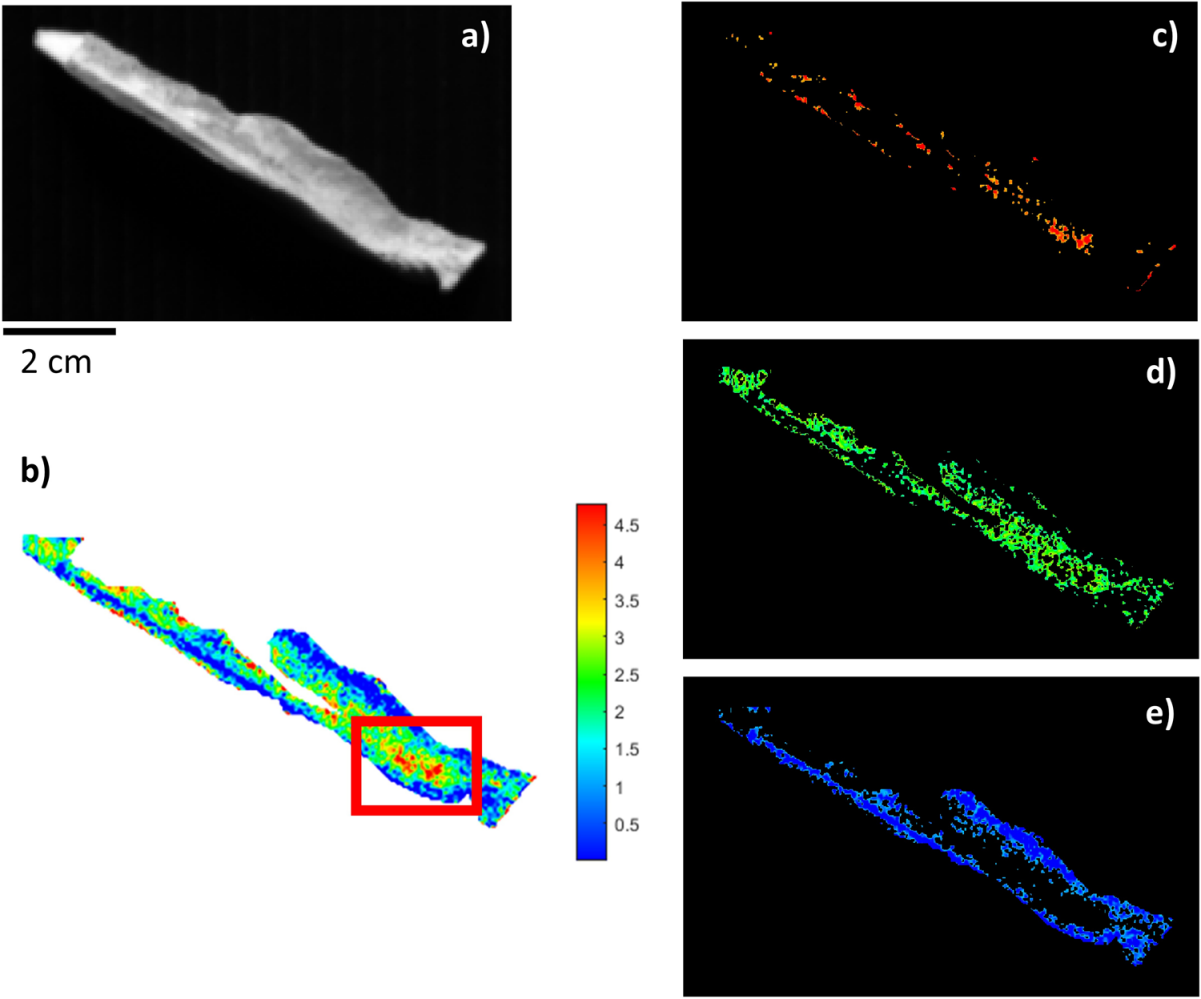
NIR-HSI results for sample BRA-6334. **a** Grey-scale total intensity image; **b** PLS map of the reference samples. The colour bar numbers refer to collagen % predicted by the PLS model. The red square area is the selected sample taken for the extraction of collagen; **c**–**e** ROIs referring to areas with different collagen concentrations (blue: low, green: mid-level, red: high) on an individually scaled map.

The analysis of the bone sample (BRA-6334) predicted a mean value of 1.85% and a standard deviation of 1.02% (Table 1 and Fig. 6). Despite the overall low amount of collagen, it was possible to distinguish the richest area in the central part of the fragment (where collagen was 2.5% ± 2.2%) from the lateral regions (where collagen was up to 0.5% ± 2.2%). This effect agreed with the typical degradation of buried bones, which usually show more marked diagenesis for the areas in direct contact with the ground. The result of the collagen subsequently extracted shows no significant differences between the estimated and actual amounts produced (Table 1), considering the root mean square error in cross-validation (RMSECV) of 2.2%.

**Fig. 7:**
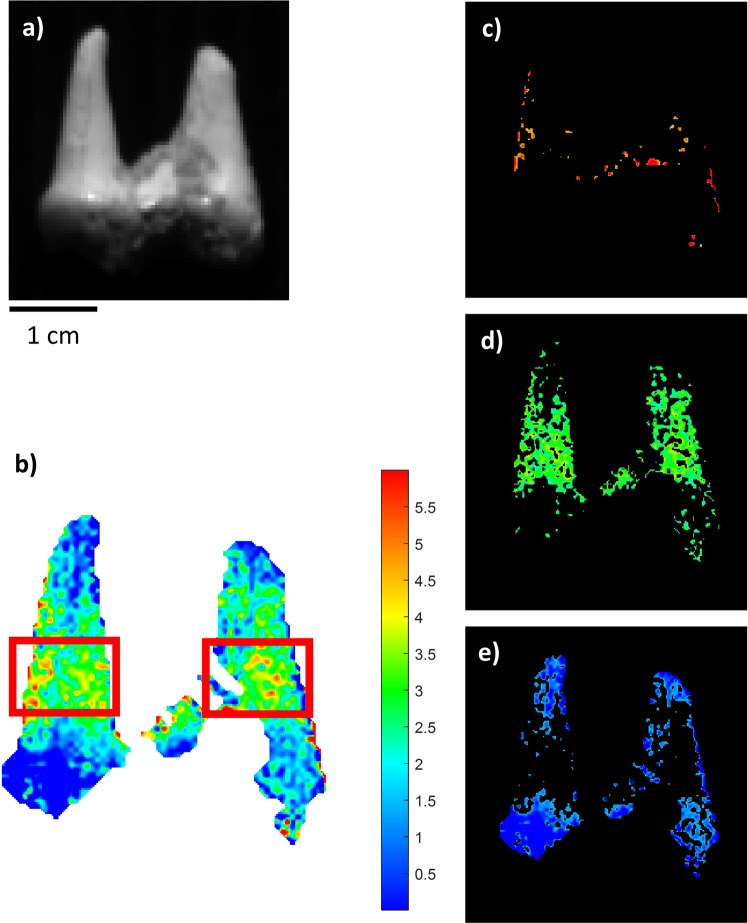
NIR-HSI results for sample BRA-6335. **a** Grey-scale total intensity image; **b** PLS map of the reference samples. The colour bar numbers refer to collagen % predicted by the PLS model. The two red squares indicate the selected samples taken for the extraction of collagen; **c**–**e** ROIs referring to areas with different collagen concentrations (blue: low, green: mid-level, red: high) on an individually scaled map.

On the tooth sample (BRA-6335), it was possible to map the collagen residues with a predicted mean value of 2.22% and a standard deviation of 1.09%, with a better-preserved area in the middle of the roots, where the estimated collagen amount was about 3% (Table 1 and Fig. 7).

Also in this case, the ROI images allowed us to easily identify the most favourable area to be submitted to sampling, strongly reducing the amount of sample needed.

BRA-6334 and BRA-6335 had already been tested for their very low amounts of collagen over several years, and these results confirmed that the NIR method is a useful way to take only a very small amount of a sample for collagen extraction in a targeted area without wasting the whole material. However, we are now confident that with a prediction of a smaller amount of collagen (∽2%) we should not attempt to destroy even a small amount of material.

## Discussion and conclusions

The sum of natural, biological and cultural events that have occurred over time is fundamental to understanding and explaining the changes we are experiencing as individuals and as a species. Bones represent the material on which to pour our investigations. Indeed, bones rich in collagen act as bridges between archaeology and the archaeological sciences. One predominant example in this article is the use of collagen for radiocarbon dating to establish the times of different cultures across the planet (e.g.^1,28–30^). However, bones, and especially collagen, can provide different kinds of information depending on the scientific method applied^5,31^.

The potential of the method proposed in the present study lies in the type and amount of information that the predictive model provides, addressing two fundamental and complementary questions for the characterisation of collagen in bones: how much and where. Thus, this experimental approach can provide quantitative information related to the average collagen content present in the whole sample submitted for investigation. The examination can be performed not only in small and localised areas (as in single-point analysis), but it can also consider the entire surface of the sample, thus producing a higher and much more significant amount of data. In addition, combining the HSI system with PLS regression allowed, on samples of ancient bones, not only to determine the overall collagen content but also to localise it at a high spatial resolution (about 30 um), obtaining quantitative chemical maps.

The strategy followed for data processing enabled the identification of three ROIs at predominant red, green and blue colours were automatically selected by defining, respectively, three numerical selection ranges of the colour values of the pixels in the resulting PLS maps. This turned out to be a further aid in visualising the points that could be more promising to collect samples for radiocarbon analysis. Moreover, the strategy followed for data processing enabled the automatic extraction of the average NIR spectrum from the three areas identified by averaging the subsets of spectra corresponding to the selected pixels.

Prescreening the whole bone is essential to target subsequent specific analyses. As far as radiocarbon is concerned, we could strategically sample bones of high patrimonial value. For example, knowing the precise amount of collagen concentrated in a precise area of the bone allows us to cut only this portion. Moreover, when the prediction of collagen shows that the bone was poorly preserved (e.g., BRA-6334 and BRA-6335), we can decide to perform a soft ^14^C pretreatment to minimise collagen loss during the extraction^32^. Generally, near-infrared radiation penetrates a few millimetres in bones^21^, revealing mainly a superficial and sub-superficial distribution of collagen.

An important issue to get as much surface scanned and analysed by the NIR-HSI is the cleaning of the surface of the bone. In fact, any excavated soil on the surface leads to an erroneous evaluation of the collagen content obtained through NIR spectroscopy because of the specific interaction between NIR radiation and concretions.

It is also worth remembering that if we cannot acquire AMS ^14^C dates on solid targets because collagen preservation is too low for routine dating, we can successfully apply the radiocarbon method using a MICADAS coupled with a gas ion source measuring the ^14^C of CO_2_ gas injected directly into the source^33^.

This situation emphasises the need to use a viable method, such as the one proposed here, to prescreen the collagen content so that high-resolution scientific techniques for analysing archaeological materials can be applied while reducing costs, time and the amount of precious materials.

Overall, this innovative and incisive combination of NIR-HSI spectroscopy prescreening and the radiocarbon method provides detailed information about the presence of collagen on archaeological bones, reducing laboratory costs by dating only materials suitable for ^14^C and increasing the number of archaeological bones that can be preserved and, therefore, exposed in museums.

In its highest sense, our cultural heritage represents the testimony of past civilisations, the sum of historical and cultural events that have occurred over time. Its value is recognised worldwide, acting as a link between different generations. Here we have shown that by using strategic coordination and effective research between spectroscopic methods and radiometric techniques to study prehistoric bones, we can provide a valuable contribution to the preservation, enhancement and protection of our historical and cultural heritage.

## Methods

### Samples

The present study involved the analysis of 44 bones from different environments ranging from the modern age to >50,000 years ago. All the necessary information about the samples is given in Table 1 and Supplementary Data 1. All the samples provided in the paper belong to the BRAVHO Radiocarbon Lab. These samples were split as follows: 15 were analysed and treated as whole pieces, and all 44 as powder samples. Four of these powder samples were pretreated for radiocarbon without using the ultrafiltration step (see Supplementary Data 1 and Table 1 in the main text).

### Predictions of the model

To develop a robust predictive model that is able to estimate the collagen content on entire bones, the training samples need to be as similar as possible to the specimens to which the model will be applied. For this reason, the best physical status of the training samples would be the entire solid bone or fragments of it; nevertheless, this type of sample is naturally characterised by the heterogeneous distribution of collagen. This, then, constitutes a hurdle when establishing a relationship between the near-infrared hyperspectral imaging (NIR-HSI) technique and the chemical determination of collagen. In fact, while NIR-HSI acquires prompt information (at a pixel level) on collagen absorption across the entire surface of the sample, the chemical determination needs to be performed on the whole fragment (or on a portion measuring about 500 mg), thus providing a global characterisation.

To overcome this limitation, two approaches were implemented in the present study at the sampling and data processing levels. At the sampling level, both solid fragments and powders were included in the calibration set; in fact, the grinding of bone fragments can be considered as a way to homogenise the samples physically. As a result, the proposed NIR imaging-based method provides operators with information comparable to that obtained from the chemical determination of the amount of collagen. Including both types of samples in the same model makes the model more robust and applicable to new samples, whether they are powders or fragments.

### NIR-imaging

For all types of samples, data were acquired from NIR-images by a push-broom system composed of a SWIR3 hyperspectral camera operating in the 1000–2500 nm spectral range at a resolution of 5.6 nm (Specim Ltd, Finland). OLES15 (Middleton Spectral Vision, Middleton, WI, USA) as the camera lens (Focal Length = 15 mm, F-number = 2.1, Transmission >82%, Minimum Working Distance = 30 cm, Field of View angle = 35.5°). NIR-images were acquired from distinct samples at the Analytical Chemistry and Chemometrics Lab in the Department of Pharmacy (DIFAR), at the University of Genova (Italy), following the procedure of ref. ^23^. The instrumental setting was characterised by three halogen lamps (35 W, 430 lm and 2900 K each) as the illumination sources and a horizontal line scanner (40 × 20 cm LabScanner, Specim Ltd, Finland) on which the samples were laid down. The system was controlled by the Lumo Scanner version 2.6 software (Specim Ltd, Finland). Before each measurement, dark (closed shutter) images and white (99% reflectance Spectralon® rod) images were then automatically recorded and stored and were used to compute the spectral reflectance value (R) for each pixel and wavelength. For the acquisition, the scan parameters were set as follows: frame rate equal to 50.00 Hz and exposure time equal to 9.00 ms. The manual focus was tuned before the scan. By scanning the entire sample surface, a complete three-dimensional HSI was created, where the first two dimensions represent the spatial information (coordinates of the planar space), while the third one is the spectral coordinate. Under the operating conditions set down for this analysis, the pixel size (side length) was equal to 0.5 mm, and the scan speed was set at 20 mm/s.

### Collagen extraction

For the present study, we selected 44 bone samples from different environments of varying ages (Supplementary Data 1). The collagen was extracted at the BRAVHO ^14^C lab in the Department of Chemistry G. Ciamician, University of Bologna (Italy), following Talamo et al.^5^ for radiocarbon dating. From all 44 samples, the collagen was extracted from bone powder, and four were subject to pretreatment without the ultrafiltration step (modified Longin as described in ref. ^5^). Of these 44, only from 15 samples the collagen extracted was from a piece (Supplementary Data 1).

Bone samples are decalcified in 0.5 M HCl at 4 °C for several hours in case of powder or for several days/weeks in case of the whole bone piece until no CO_2_ effervescence is observed. 0.1 M NaOH is added for 30 min to remove humic acids, followed by 0.5 M HCl for 15 min. The resulting solid is gelatinised in HCl pH 3 in a heater block at 75 °C for 20 h. The gelatin is then filtered with an Eeze-Filter™ (Elkay Laboratory Products (UK) Ltd.) to remove small (>80 µm) particles. The gelatin is then ultrafiltered (Sartorius Vivaspin Turbo 15 with a polyethersulfone membrane and 30 kDa MWCO). Prior to use, the filters are carefully cleaned. The >30 kDa fraction was frozen for 24 h and then lyophilised for 48 h and weighed to determine the % of collagen.

### Multivariate data processing

After the first step involving the NIR images of the bone samples (both powders and fragments), the background was removed according to manual masking. Spectral profiles were then extracted from pixels belonging to the samples and examined to confirm the characteristic NIR absorptions of collagen. To obtain a full characterisation of each sample, the mean profile was computed for the powder samples, while the median profile was chosen as a better description of the fragments. In fact, fragments are characterised by a higher inhomogeneity, and the median is a more robust descriptor.

Spectral mean/median profiles were pre-processed by applying the standard normal variate transform (SNV) as a scatter-corrective pre-processing^34^, together with a second-order Savitzky–Golay derivative (15-datapoint window size, third-degree polynomial). The spectra range was also reduced by removing the wavelengths from 1000 to 1060 nm, due to the presence of the low signal-to-noise ratio that characterised the initial part of the spectral range and related to detector efficiency. The pre-processing strategy applied ensured the removal of the baseline shift and drift and unwanted global intensity effects, thus the differentiation among the areas classified as blue, green or red is due to variations in band intensity and shape.

The calibration set of 59 bone samples in total, including the 44 powders and 15 fragments, were used to develop the model. The data were pretreated by column mean-centring. The optimal complexity (number of latent variables) of the PLS models was selected by applying a cross-validation scheme to the training set data with five cancellation groups and choosing the number of latent variables (LV) corresponding to the model characterised by the lowest root mean square error of cross-validation (RMSECV). Data analysis was performed under MATLAB environment (v. 2019b, The MathWorks, Inc., Natick, MA, USA).

### Reporting summary

Further information on research design is available in the Nature Portfolio Reporting Summary linked to this article.

## Supplementary information

Description of Additional Supplementary File

Supplementary Data 1

Reporting Summary

## Data Availability

The data supporting the findings in this study are available within the manuscript and Supplementary Data 1. The datasets generated during and/or analysed during the current study are available from the corresponding author on reasonable request.
