# Supplementary material for: Near-infrared hyperspectral imaging to map collagen content in prehistoric bones for radiocarbon dating
Source: Commun Chem. 2023 Apr 11;6:54. doi: 10.1038/s42004-023-00848-y (PMC10090164; doi:10.1038/s42004-023-00848-y)
Supplement: Supplementary file 1 — Description of Additional Supplementary File [file 42004_2023_848_MOESM1_ESM.pdf]

Description of Additional Supplementary File

File name: Supplementary Data 1

Description: The 44 archaeological samples were used to build and validate the model and obtain quantitative data. All 44 samples were drilled to obtain powder samples, and only 15 samples were cut and pretreated as whole pieces.
